# Detection of *Sclerotinia* Stem Rot on Oilseed Rape (*Brassica napus* L.) Leaves Using Hyperspectral Imaging

**DOI:** 10.3390/s18061764

**Published:** 2018-06-01

**Authors:** Wenwen Kong, Chu Zhang, Feng Cao, Fei Liu, Shaoming Luo, Yu Tang, Yong He

**Affiliations:** 1 School of Information Engineering, Zhejiang A & F University, Hangzhou 311300, China; wwkong16@zafu.edu.cn; 2 College of Biosystems Engineering and Food Science, Zhejiang University, Hangzhou 310058, China; chuzh@zju.edu.cn (C.Z.); caofeng0702@163.com (F.C.); fliu@zju.edu.cn (F.L.); 3 Key Laboratory of Spectroscopy Sensing, Ministry of Agriculture, Hangzhou 310058, China; 4 College of Automation, Zhongkai University of Agriculture and Engineering, Guangzhou 510225, China; smluo@gdut.edu.cn

**Keywords:** hyperspectral imaging, oilseed rape, *Sclerotinia* stem rot, variable selection, discriminant methods, calibration transfer

## Abstract

Hyperspectral imaging was explored to detect *Sclerotinia* stem rot (SSR) on oilseed rape leaves with chemometric methods, and the influences of variable selection, machine learning, and calibration transfer methods on detection performances were evaluated. Three different sample sets containing healthy and infected oilseed rape leaves were acquired under different imaging acquisition parameters. Four discriminant models were built using full spectra, including partial least squares-discriminant analysis (PLS-DA), support vector machine (SVM), soft independent modeling of class analogies (SIMCA), and k-nearest neighbors (KNN). PLS-DA and SVM models were also built with the optimal wavelengths selected by principal component analysis (PCA) loadings, second derivative spectra, competitive adaptive reweighted sampling (CARS), and successive projections algorithm (SPA). The optimal wavelengths selected for each sample set by different methods were different; however, the optimal wavelengths selected by PCA loadings and second derivative spectra showed similarity between different sample sets. Direct standardization (DS) was successfully applied to reduce spectral differences among different sample sets. Overall, the results demonstrated that using hyperspectral imaging with chemometrics for plant disease detection can be efficient and will also help in the selection of optimal variable selection, machine learning, and calibration transfer methods for fast and accurate plant disease detection.

## 1. Introduction

Oilseed rape is one of the major oil crops in the world, and diseases represent severe threats to oilseed rape plants. *Sclerotinia sclerotiorum* is the major disease of oilseed rape in all major growing regions, including China. *Sclerotinia sclerotiorum* can affect the leaf, stem, pod, and flowers of oilseed rape plants. In general, *Sclerotinia sclerotiorum* can cause 0–20% of yield loss every year and the severe situation of *Sclerotinia sclerotiorum* infection in China can reach 80% of yield loss [1]. The timely and accurate detection of *Sclerotinia sclerotiorum* is urgent in precision oilseed rape disease management. However, traditional methods for accurate plant disease detection are DNA-, RNA-, and serological-based [2,3,4,5], which are time-consuming, expensive, require a professional’s operation, and are not suitable for rapid, online, large-scale detection. Thus, rapid and accurate detection methods are needed.

Hyperspectral imaging at the visible/near-infrared range has been widely used in plant disease detection because spectral and spatial information are provided simultaneously. Hyperspectral imaging has great potential for the rapid and accurate online, large-scale detection of plant diseases. Infected and healthy organs and plants can be revealed by spectral differences. In recent studies, the use of visible/near-infrared spectroscopy and the corresponding hyperspectral imaging in plant disease detection has been examined [6,7,8,9,10,11,12].

The management of large amounts of spectral data is essential and crucial in visible/near-infrared spectroscopy and the corresponding hyperspectral imaging. Optimal wavelength selection methods and machine learning methods have been widely applied to spectral data for quantitative analysis [13,14,15,16,17,18,19]. The objective of applying optimal wavelength selection methods and machine learning methods to visible/near-infrared spectroscopy and hyperspectral imaging is to identify the optimal solution for data interpretation, to build optimal calibration models for quantitative analysis, and to explore the feasibility of large-scale, real-world application.

An important problem to be addressed is that only on a single sample set with a limited number of samples is used to explore the feasibility of detecting diseases [6,7,8,9,10,11,12]. Additionally, the future trend is to develop portable devices or vehicle-based devices with simple, accurate, and robust models for real-world application. Thus, the selection of optimal wavelengths and detection models with great universality are essential. However, whether the selection of optimal wavelengths and detection models using a single sample set with a limited sample number work well on other sample sets remains to be determined.

Different users using the same type of hyperspectral imaging for the same purpose commonly acquire hyperspectral images from different acquisition conditions. The direct application of one established model on different sample sets is difficult because of the differences between the predicted sample sets and the calibrated sample set. However, establishing models for each sample set will increase the costs and limit the real-world application. Calibration transfer is a widely used technique to solve this problem and the spectra of sample sets acquired from different times or conditions are transferred and used for prediction by calibration models using the spectra of a predefined standard sample set to remove the spectra differences [20]. Compared with establishing individual models on each sample set, the calibration transfer method is more economical and cost-effective. Calibration transfer has been studied in spectral data analysis. In plant disease detection, calibration transfer can help to extend the application of established models, particularly when detection is conducted under different conditions. Thus, the calibration transfer is of great value in moving this research into real-world calibration.

In previous studies, mid-infrared spectroscopy was used to detect *Sclerotinia* stem rot (SSR) on oilseed rape leaves [21], and hyperspectral imaging to detect SSR on oilseed rape stems [22]. Pixel-wise spectra-based analyses and modeling showed the efficiency of using hyperspectral imaging to detect and locate SSR on oilseed rape stems [22]. However, the influences of optimal wavelengths selection, discriminant models, and different imaging conditions were not further studied. The aims of this study are as follows: (1) to investigate the possibility and accuracy of detecting *Sclerotinia* stem rot (SSR) on oilseed rape leaves using hyperspectral imaging on three different sample sets; (2) to compare and select the optimal modeling methods and optimal wavelength selection methods for a robust model; (3) to explore the model transfer methods to detect SSR with different time stages on oilseed rape leaves using direct standardization (DS).

## 2. Materials and Methods

### 2.1. Sample Preparation

In this study, three experiments using the same oilseed rape cultivar (cv. ZS758) were conducted to obtain three sample sets. The first experiment was conducted at the seedling stage of oilseed rape in 2013. The *Sclerotinia sclerotiorum* were cultured on a potato dextrose agar medium. When four or five expanded leaves were on each plant, 90 oilseed plants were transplanted into flower-pots in the greenhouse. When the plants adjusted to the environment, mycelial pellets were placed on the plant leaves and each leaf received two mycelial pellets symmetrically placed along the main vein. Forty-five infected plants were placed in a controlled environment in which the main parameters were set as follows: a temperature of 25 °C and a relative humidity of 85%. The healthy plants were placed under the same conditions in a separate room. Forty-eight hours later, 45 infected leaves and 45 healthy leaves were collected.

The second and the third experiments were conducted in 2015, with the same procedure applied as that used for the experiment in 2013. A total of 200 plants were transplanted into flower-pots in the greenhouse. For the second experiment, 150 leaves on 60 plants were used for inoculation with two mycelial pellets placed symmetrically along the main vein and the plants were placed in a controlled environment in which the main parameters were set as follows: a temperature of 20 °C and a relative humidity of 80%. Thirty healthy plants were kept in the same environment, in an isolated room. After 72 h, 60 infected leaves and 60 healthy leaves were collected. For the third experiment, 60 of the remaining plants were used for inoculation and 30 were used as healthy plants. After 72 h, 60 infected leaves and 60 healthy leaves were collected. The random selection method was used to obtain the calibration set (248 samples) and the prediction set (82 samples).

### 2.2. Hyperspectral Images Acquisition and Correction

The hyperspectral imaging system was an assembled system introduced in a previous study [23]. For the first experiment, the distance between the sample and the lens, the exposure time of the camera, and the moving speed of the sample plate were set to 360 mm, 0.05 s, and 2.05 mm/s, respectively. For the second and third experiments, the three parameters were adjusted to 400 mm, 0.09 s, and 2.7 mm/s, respectively. After the image acquisition, an image correction procedure was conducted based on the methods in a previous study [23].

### 2.3. Spectra Extraction

Each corrected hyperspectral image contained two or three leaves. First, the image containing one leaf was cut from the corrected image, then the leaves were isolated from the background and the entire leaf region was defined as the region of interest (ROI). The spectrum of all pixels within the ROI were extracted and their average value was regarded as the spectrum of the sample.

### 2.4. Chemometric Methods

#### 2.4.1. Partial Least Squares-Discriminant Analysis

Partial least squares-discriminant analysis (PLS-DA) is a widely used supervised pattern recognition method. PLS-DA conducts the regression procedure using the spectral data as X and the integers representing categories as Y. The linear relationship between X and Y is explored. The outputs of the prediction are real numbers with decimals. Thus, the threshold value is applied on the prediction values to determine the category to which the samples belong. Generally, the threshold value is set to 0.5 [24]. If the absolute value of the difference between the reference value and the prediction value is lower than 0.5, the sample is correctly classified, otherwise it is misclassified. For the PLS-DA model, the number of latent variables (LVs) should be determined for optimal PLS-DA models. In this study, leave-one-out cross-validation was used to determine the optimal number of LVs.

#### 2.4.2. Support Vector Machine

Support vector machine (SVM) is a supervised pattern recognition method. SVM maps the original sample data into a higher-dimensional space by kernel functions and constructs a hyperplane or a set of hyperplanes in the higher dimensional space to maximize the distance between the nearest samples of two classes. The radial basis function (RBF) is a commonly used kernel function in SVM and can provide good performances in spectral analyses [25]. The penalty coefficient (C) of the SVM model and the kernel width (g) of the kernel function must be determined. In this study, the SVM models were built using five-fold cross-validation. The optimal combination of (C, g) was determined by a grid-search procedure and the ranges of C and g were both 2^−8^–2^8^.

#### 2.4.3. Soft Independent Modeling of Class Analogies

Soft independent modeling of class analogies (SIMCA) is a supervised pattern recognition method based on principal component analysis (PCA). SIMCA first applies PCA to each class and then the optimal number of principal components (PCs) is determined for each class. The samples are then classified based on the scores of the optimal PCs [25]. For the computation of PCA, leave-one-out cross-validation was implemented and the first 20 PCs were explored. SIMCA models were built and compared using different numbers of PCs.

#### 2.4.4. k-Nearest Neighbor

The k-nearest neighbor (KNN) method is a simple and effective pattern recognition method. In KNN, the sample distances determine the k-nearest neighbors, and the sample class is determined by a majority vote by the classes of the k-nearest neighbors. The number of the nearest neighbors (k) is the main factor influencing the model performance. The selection of k is important for KNN [26]. In this study, the number of nearest neighbors was explored from 3 to 10.

### 2.5. Variable Selection Methods

#### 2.5.1. Second Derivative Spectra

Second derivative is a commonly used spectral preprocessing method. Second derivative spectra can increase the spectral resolution and suppress the background. The extracted spectrum consists of broad and heavily overlapped features. The resolution enhancement in the second derivative spectra depends on the fact that it can highlight the spectral peaks and identify overlapping spectral peaks. Differences between the spectral peaks can be identified for sample differences. The spectral peaks with obvious differences are selected as the optimal wavelengths [27].

#### 2.5.2. Principal Component Analysis (PCA) Loadings

PCA transforms the original data into new orthogonal variables (PCs), with the first few PCs containing the most useful information. The loadings indicate the relative importance of the wavelengths within each PC. Generally, the loadings of the first few PCs are used for optimal wavelength selection [14].

#### 2.5.3. Successive Projection Algorithm

Successive projection algorithm (SPA) is an efficient variable selection method to select variables with minimum redundancy and collinearity. The SPA projects one wavelength variable on the other wavelength variables and the variable with the maximum projections is selected into the candidate subset of the optimal wavelengths. Then, the calibration models are built on different variables within the candidate subset and the optimal wavelengths are determined by the model performances [28].

#### 2.5.4. Competitive Adaptive Reweighted Sampling

Competitive adaptive reweighted sampling (CARS) is an efficient variable selection method based on regression coefficients in a multivariate linear regression model (in this study, PLS-DA). The variable importance is evaluated by the absolute value of the regression coefficient and the variables with higher absolute values are selected.

Monte-Carlo sampling is used to select a fixed ratio of samples in the calibration set to build calibration models and the remaining samples are used for prediction. Exponentially decreasing function (EDF) and adaptive reweighted sampling (ARS) are used to determine the candidate subsets. The calibration models are then built using the candidate subsets and the subset corresponding to the minimum root mean square error of cross validation (RMSECV) is selected as the optimal wavelength [29].

### 2.6. Calibration Transfer Method

Direct standardization (DS) is an efficient calibration transfer method [20]. The general concept of DS is to form a transformation matrix to transfer the spectra between different spectral datasets. The general procedure of DS can be summarized as follows:

(1) Given two known spectra datasets **X** and **S**, Equation (1) is used to calculate the transformation matrix **F**:

**S** = **XF** (1)

(2) The transformation matrix can be expressed as:

**F** = **X**^+^**S** (2)

where **X**^+^ is the generalized inverse matrix of **X**.

(3) After obtaining **F**, the transferred spectra can be calculated by Equation (2). The transferred spectra can be predicted by the models established by the source spectra (measured the same way as **S**).

### 2.7. Model Evaluation and Software

The classification accuracy of the calibration and prediction sets were considered as the main parameters of the model evaluation in this study. PLS-DA and the second derivative were conducted on Unscrambler 10.1 (CAMO AS, Oslo, Norway); PCA, KNN, SVM, SIMCA, SPA, and CARS were conducted on Matlab R2014b (The Math Works, Natick, MA, USA).

## 3. Results

### 3.1. Spectral Profile

**Figure 1:**
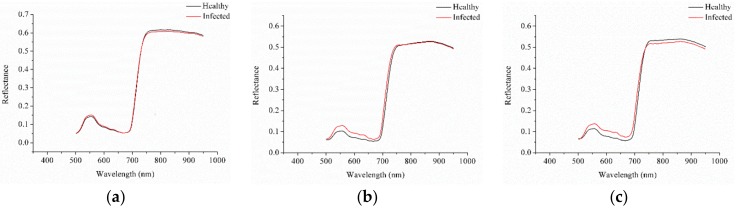
The average spectra of healthy and infected leaves of sample sets 1 (**a**), 2 (**b**) and 3 (**c**).

Because of the system and environment noises, only the spectra in the range of 500.98–950.13 nm were analyzed. Figure 1 shows the average spectra of the healthy and infected leaves of sample sets 1, 2, and 3. The typical reflectance spectra of green plants could be found in the three sample sets.

The average spectra of the healthy and infected leaves of sample sets 1, 2, and 3 showed no obvious differences and only slight differences in the spectral reflectance value could be observed. Sample set 1 showed higher reflectance values than sample sets 2 and 3 because of the different conditions for the acquisition of the hyperspectral images. The spectra were preprocessed using moving average smoothing with seven smoothing points. To build the detection models, the samples of each sample set were randomly divided into two sets and the ratio of the calibration set to the prediction set was 3:1.

### 3.2. Full Detection Model

The PLS-DA, SVM, KNN, and SIMCA models were first built using the full spectra of the three sample sets. In the selection of the optimal models, the models with a better prediction accuracy were chosen as the optimal models. All the models obtained acceptable results, indicating the feasibility of using the full spectra to detect SSR on oilseed rape leaves.

The discriminant models of the three different sample sets showed that the performance of sample set 1 was relatively low, whereas sample sets 2 and 3 obtained relatively better performances. For sample sets 1, 2, and 3, the models with the highest classification accuracy were the PLS-DA, SVM, and PLS-DA models, respectively. The performances of the discriminant models showed different regulations. Fox example, SIMCA performed better than the SVM model on sample set 1, and the SIMCA models on sample sets 2 and 3 performed worse than the SVM models.

For sample set 1, the classification accuracies among the different models were obvious. For sample set 2, the classification accuracies among the different models were small. For sample set 3, the classification accuracies among the different models were the smallest. The use of discriminant models showed influences on the detection performance. However, the optimal detection models could not be simply determined. Overall, the use of sample sets with limited sample numbers to determine the optimal discriminant models was confined to the sample sets, and whether the selected optimal models were universal could not be evaluated. The results in Table 1 showed the feasibility and effectiveness of the different discriminant models in oilseed rape SSR detection.

### 3.3. Optimal Wavelength Selection

Optimal wavelength selection aims to select wavelengths that carry the most information with minimum collinearity and redundancy. The selected optimal wavelengths were used to reduce the model complexity, simplify the models, and to reduce the computation task. In this study, the selected optimal wavelengths contributed the most to SSR detection on oilseed rape leaves.

Second derivative spectra, PCA loadings, SPA, and CARS were used to select the optimal wavelengths. PCA loadings and second derivative spectra selected the optimal wavelengths based on the spectral information. The optimal wavelengths selection by SPA and CARS were based on the evaluation of the models and wavelengths that fitted with the optimal evaluation model performances were selected.

**Figure 2:**
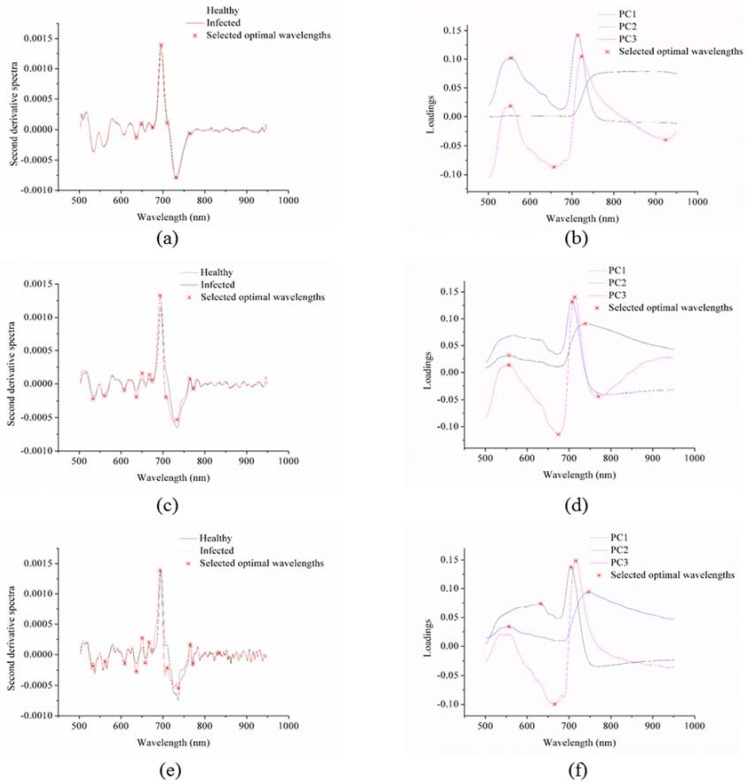
The optimal wavelength selection by the second derivative spectra of the sample sets 1, 2, and 3 ((**a**,**c**,**e**), respectively) and the principal component analysis (PCA) loadings of the three sets ((**b**,**d**,**f**), respectively).

The second derivative method preprocessed the average spectra of the healthy and infected leaves of the three sample sets, as shown in Figure 2a,c,e, respectively. As shown in Table 2, the selected optimal wavelengths of the three sample sets showed great similarity, with only a slight shift. The primary differences were in the number of selected wavelengths.

Table 2 shows the optimal wavelengths selected by the PCA loadings, second derivative spectra, SPA, and CARS for the three sample sets.

The first three PCs of each sample set explained more than 99% of the total variance, and the loadings of the first three PCs of sample sets 1, 2 and 3 are shown in Figure 2b,d,f, respectively. The loading curves of the three sample sets showed certain regulations. Some of the selected optimal wavelengths were similar.

The second derivative spectra and the PCA loadings were manually applied as optimal wavelength selection methods. The optimal wavelengths were selected by the spectral differences or internal information.

The optimal wavelengths selected by CARS were different in the different sample sets and the optimal wavelengths selected by the SPA were also different in the different sample sets.

Therefore, the optimal wavelengths selected by the four methods and the corresponding numbers of the wavelength were different. The selected optimal wavelengths are shown in Table 2.

### 3.4. Discriminant Models Using Optimal Wavelengths

Table 1 shows that the PLS-DA and SVM models showed better results for the three sample sets; therefore, these two methods were used to build discriminant models with optimal wavelengths. The results are shown in Table 3.

For sample set 1, the results for the PLS-DA models were worse using optimal wavelengths than those using full spectra. The SVM models using optimal wavelengths selected by SPA and CARS showed better results than the models using full spectra. For sample sets 2 and 3, the PLS-DA and SVM models using optimal wavelengths obtained similar results than those using full spectra.

The objective of the optimal wavelength selection is to reduce data collinearity and redundancy, to simplify the models, and to reduce the computation tasks while maintaining or improving the model performance. In this study, three sample sets containing healthy and infected leaves were used. As shown in Table 2, only the optimal wavelengths were selected by the second derivative spectra and the PCA loadings showed a similarity between the different sample sets; within one sample set, the optimal wavelengths selected by the four different methods were different. As shown in Table 3, the discriminant models using the selected optimal wavelengths showed acceptable results.

The optimal wavelengths selected by one sample set were also applied to the other sample sets and the results are shown in Table 4. The discriminant models of one sample set using the optimal wavelengths selected by the other sample sets all obtained acceptable results, and some models showed similar or even better results than the models of the sample set using the optimal wavelengths selected from the sample set itself.

### 3.5. Model Transfer

Model transfer is important in spectral data analysis and a key factor for the real-world application of spectroscopy-related techniques. Sample sets 2 and 3 were examined under the same parameters of the hyperspectral imaging system and the spectra were not identical. Sample set 1 was examined under different image acquisition parameters from those of sample sets 2 and 3. Figure 1 shows the differences of the spectra of the different sample sets. The model transfer for the oilseed rape SSR detection under different image acquisition conditions was explored. DS was applied to conduct transfer calibrations. Figure 1 shows the differences between the spectra of healthy samples and the spectra of infected samples; thus, the transfer procedure was conducted on the spectra of healthy samples and the spectra of infected samples. As shown in Table 1, overall, the discriminant models of sample set 3 obtained relatively better results than those of the other sets; thus, sample set 3 was used as the standard sample. To transform sample set 1, 34 healthy samples and 34 infected samples from the calibration set of sample set 3 were used as standard samples. To transform sample set 2, 45 healthy samples and 45 infected samples from the calibration set of sample set 3 were used as standard samples. The transferred and untransferred samples of sample sets 1 and 2 were predicted by the SVM model built using the calibration set of sample set 3. The results are shown in Table 5.

As shown in Table 5, the prediction results of the untransferred samples of sample set 1 were unsatisfactory, whereas the prediction results of the untransferred samples of sample set 2 were satisfactory with a prediction accuracy of 90.83%. As shown in Figure 1, the spectral reflectance of sample set 1 was higher than those of sample sets 2 and 3, and the image acquisition parameters of sample set 1 were different from those of sample sets 2 and 3. Sample sets 2 and 3 were acquired under the same image acquisition parameters, which explained why the prediction accuracy of the untransferred samples of sample set 2 exceeded 90%. The prediction accuracy of the transferred samples of sample set 1 improved greatly, increasing from 47.78% to 94.44%. The prediction accuracy of the transferred samples of sample set 2 improved slightly.

Overall, the results were promising for the calibration transfer to reduce the spectra differences caused by different image acquisition parameters and conditions. Therefore, the use of calibration transfer to overcome the spectral differences caused by different factors could help hyperspectral imaging used in reality.

## 4. Discussion

The fast and accurate online detection of plant diseases is a crucial component of precision agriculture and is necessary to help guide farmers and researchers in the precision control of plant diseases to reduce losses in yield and quality, which can also help reduce the use of pesticides.

In this study, hyperspectral images of oilseed rape leaf samples were acquired. The spectral information was extracted and analyzed using the entire leaf area as the region of interest (ROI). Chemometric methods, including variable selection and machine learning methods, have been widely used in plant disease detection using visible/near infrared spectroscopy and hyperspectral imaging [6,7,8,9,10,11,12,30,31,32]. Exploring the spectral differences between the healthy and infected samples, the samples infected by different diseases, and the samples with different disease infection severities was the initial goal of using visible/near-infrared spectroscopy and hyperspectral imaging. Researchers attempted to identify optimal wavelengths [6,9,10,11] or vegetation indices [33,34,35,36] to reduce the amount of data and computation tasks in addition to exploring optimal models for the detection of diseases.

In this study, the results of the optimal wavelength selection showed that the optimal wavelengths selected by different methods were different. However, the discriminant models using the optimal wavelengths showed acceptable results. In the studies of Zhang, Jiang, Liu, and He [37] as well as those of Balabin and Smirnov [38], 10 and 16 optimal wavelength selection methods were used and the selected optimal wavelengths were rarely the same when acquired by different methods. In this study, four different methods were used to select the optimal wavelengths and, in total, more than 30 wavelengths were selected from the original dataset. Thus, most of the variables from the original dataset could be selected by different methods.

The four methods used in this study included two different types: the first type focused on using only the spectral information for manual selection and included second derivative spectra and PCA loadings; the second type focused on optimal wavelengths selection by a two-step selection (candidate subsets selection and final optimal wavelengths by evaluation function) and included SPA and CARS. The second type can be influenced by the selection criteria of the candidate subsets and the use of the evaluation function. In this study, the optimal wavelengths selected by SPA and CARS of the three sample sets were different, whereas the optimal wavelengths selected by the PCA loadings and second derivative spectra showed similarity. Zhang et al. [21] also used mid-infrared spectroscopy to identify SSR on oilseed rape leaves and the results obtained by the second derivative spectra showed similar optimal wavelengths between the two sample sets. Kong et al. [22] used hyperspectral imaging to detect SSR on oilseed rape stems and the optimal wavelengths selected by the PCA loadings and second derivative spectra in different sample sets showed similarities.

**Figure 3:**
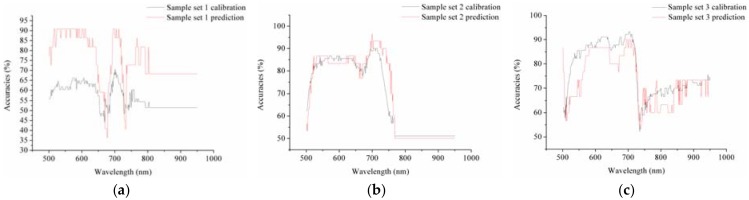
The discriminant results of the SVM models using a single wavelength band of sample set 1 (**a**); set 2 (**b**); and set 3 (**c**).

The models developed on different optimal wavelengths selected by different methods all showed acceptable results. This requires further study to provide an explanation. Therefore, the discriminant ability of each wavelength of each sample set was explored. The results of the calibration and prediction set by SVM models using single wavelengths are shown in Figure 3. For the three different sample sets, wavelengths between 500 and 770 nm showed higher discriminant accuracies, with a slight depression in the curve between 650 and 700 nm. As shown in Table 2, most of the selected wavelengths were in the range of 500–770 nm. The selected wavelengths between 500 and 750 nm were attributed to the leaf pigments that included chlorophyll and carotenes, which was confirmed in a previous study [39]. Besides that, the selected wavelengths between 900 and 970 nm were attributed to the water status of plants [40]. From the perspective of statistical analysis, the combination of the wavelengths within the range would likely obtain good performances. A different number of optimal wavelengths was selected by the same method on different sample sets and the use of more optimal wavelengths may obtain better results. This might be the reason why the optimal wavelengths selected by one sample set also obtained similar or better results in the other sample sets than the optimal wavelengths selected by the sample sets themselves. From Figure 3 and Table 2 and Table 3, it could be found that the combination of wavelengths selected in the range of wavelengths with high classification accuracies could obtain acceptable results on all sample sets, even when the combined wavelengths were totally different from the optimal wavelengths selected by the optimal wavelength selection methods from the sample set itself.

In order to compare the performance of different calibration strategies, a combined sample set was applied for model development. In this model, the calibration set (248 samples) was the composite of the calibration samples in sample sets 1, 2, and 3. The prediction set (82 samples) was the composite of the prediction samples in sample sets 1, 2, and 3. The prediction results by PLS-DA, SVM, KNN, and SIMCA are shown in Table 6. Compared with Table 1, Table 3, Table 4 and Table 5, a similar model effectiveness was achieved and the optimal prediction accuracy was over 95%. Hence, the strategy of the model transfer method and combined sample were both acceptable in this study.

The discriminant models used in this study all showed acceptable results, although their performances differed. Many methods were used to build the discriminant models and, therefore, the discriminant models should have great universality with guaranteed performances. However, the discriminant performances varied due to different discriminant models. Balabin, Safieva, and Lomakina [41] explored the differences among the different discriminant methods for gasoline classification. In this study, the PLS-DA and SVM models showed better performances than the KNN and SIMCA models based on the results of the three sample sets.

Many factors influence the acquired images and, therefore, importantly, the hyperspectral image acquisition conditions are not all the same. The major factors are the image acquisition parameters and conditions, particularly when these systems are applied in the field. The calibration transfer can provide an alternative to overcome the problems caused by different image acquisition parameters and conditions. As shown in Table 5, the use of calibration transfer for plant disease detection is feasible.

To bring the fast, accurate, and easy-to-operate hyperspectral imaging technique to real-world online applications, the accurate and cautious selection of optimal wavelengths and detection models with great universality should be rigorously studied, in addition to further studies on the role of calibration transfer.

## Figures and Tables

**Table 1:** The results of discriminant models using full spectra of three different sample sets.

Sample	Models	Parameters *	Calibration Accuracy (%)	Prediction Accuracy (%)
Set 1	PLS-DA	16	100.00	90.91
	SVM	(256, 0.3229)	79.42	81.82
	KNN	4	66.17	59.09
	SIMCA	(10, 10)	80.88	95.95
Set 2	PLS-DA	6	96.67	96.67
	SVM	(256, 1.7411)	100.00	100.00
	KNN	4	91.11	100.00
	SIMCA	(10, 10)	90.00	90.00
Set 3	PLS-DA	15	100.00	100.00
	SVM	(48.5029, 1.7411)	98.89	100.00
	KNN	3	93.33	93.33
	SIMCA	(9, 9)	95.56	100.00

* The parameters indicate the parameters of each model; the parameters for partial least squares-discriminant analysis (PLS-DA), support vector machine (SVM), k-nearest neighbors (KNN), and soft independent modeling of class analogies (SIMCA) are the optimal number of latent variables, (C, g), the number of nearest neighbors, and number of principal components (PCs) of each class, respectively.

**Table 2:** The optimal wavelengths selected by the PCA loadings, second derivative spectra, successive projections algorithm (SPA), and competitive adaptive reweighted sampling (CARS) for the three sample sets.

Sample	Methods	No. *	Wavelengths (nm)
Set 1	Second derivative	7	636, 649, 676, 696, 710, 732, 764
	PCA loading	5	553, 657, 714, 723, 924
	SPA	14	668, 673, 550, 570, 681, 831, 521, 770, 663, 690, 932, 638, 650, 511
	CARS	16	550, 554, 559, 580, 589, 621, 628, 643, 663, 664, 852, 871, 901, 916, 945, 950
Set 2	Second derivative	12	533, 560, 608, 636, 650, 668, 674, 693, 707, 734, 764, 773
	PCA loading	6	557, 673, 707, 714, 739, 770
	SPA	5	730, 782, 702, 558, 950
	CARS	16	629, 634, 636, 649, 762, 771, 798, 828, 837, 870, 875, 900, 908, 926, 940, 950
Set 3	Second derivative	13	533, 562, 609, 636, 650, 658, 667, 674, 693, 710, 737, 765, 771
	PCA loading	5	557, 633, 705, 716, 747
	SPA	16	813, 833, 896, 767, 867, 507, 908, 513, 930, 535, 501, 743, 557, 696, 649, 707
	CARS	9	558, 564, 571, 604, 657, 662, 766, 809, 833

* No. refers to the number.

**Table 3:** The results of PLS-DA and SVM models using optimal wavelengths of three sample sets.

Model	Sample	Methods	Parameters	Calibration Accuracy (%)	Prediction Accuracy (%)
PLS-DA	Set 1	PCA loadings	5	76.47	81.82
		Second derivative	7	77.94	68.18
		CARS	14	100.00	72.73
		SPA	14	100.00	82.61
	Set 2	PCA loadings	5	97.78	96.67
		Second derivative	5	93.33	96.67
		CARS	12	100.00	100.00
		SPA	5	93.33	96.67
	Set 3	PCA loadings	5	97.78	100.00
		Second derivative	5	100.00	100.00
		CARS	9	100.00	96.67
		SPA	12	100.00	100.00
SVM	Set 1	PCA loadings	(84.4485, 27.8576)	72.06	77.27
		Second derivative	(0.5743, 256)	69.12	90.91
		CARS	(256,84.4485)	91.18	81.82
		SPA	(147.0334, 256)	94.12	90.91
	Set 2	PCA loadings	(147.0334, 256)	98.89	96.67
		Second derivative	(147.0334, 84.4485)	97.78	100.00
		CARS	(256, 27.8576)	90.00	93.33
		SPA	(256, 147.0334)	100.00	96.67
	Set 3	PCA loadings	(27.8576, 84.4485)	97.78	100.00
		Second derivative	(256, 16)	100.00	100.00
		CARS	(5.2780, 27.8576)	91.11	83.22
		SPA	(5.2780, 84.4485)	98.89	100.00

**Table 4:** The discriminant results of the sample sets using the optimal wavelengths selected by the other sample sets.

Model	Sample/Wavelength	Methods	Parameters	Calibration Accuracy (%)	Prediction Accuracy (%)
PLS-DA	Set 1/OPW 2 ^a^	PCA loadings	4	77.94	77.27
		Second derivative	11	98.53	90.91
		CARS	14	98.53	81.82
		SPA	5	85.29	81.82
	Set 1/OPW 3 ^b^	PCA loadings	4	75.00	77.27
		Second derivative	11	98.53	86.36
		CARS	7	95.59	77.27
		SPA	11	94.12	86.36
	Set 2/OPW 1 ^c^	PCA loadings	2	85.56	90.00
		Second derivative	4	88.89	90.00
		CARS	3	84.44	90.00
		SPA	3	82.22	90.00
	Set 2/OPW 3	PCA loadings	5	94.44	96.67
		Second derivative	5	92.22	96.67
		CARS	2	82.22	80.00
		SPA	7	98.89	96.67
	Set 3/OPW 1	PCA loadings	4	95.56	90.00
		Second derivative	4	92.22	86.67
		CARS	11	98.89	100.00
		SPA	9	100.00	96.67
	Set 3/OPW 2	PCA loadings	5	94.44	90.00
		Second derivative	5	100.00	96.67
		CARS	11	100.00	100.00
		SPA	3	94.44	86.67
SVM	Set 1/OPW 2 ^a^	PCA loadings	(1.7411, 84.4485)	69.12	90.91
		Second derivative	(256, 147.0334)	89.71	86.36
		CARS	(256, 48.5029)	83.82	81.82
		SPA	(256, 147.0334)	88.24	86.362
	Set 1/OPW 3 ^b^	PCA loadings	(16, 84.4485)	72.06	86.362
		Second derivative	(256, 147.0334)	89.71	81.82
		CARS	(256, 147.0334)	80.88	68.18
		SPA	(256, 9.1896)	79.41	81.82
	Set 2/OPW 1 ^c^	PCA loadings	(256, 9.1896)	90.00	93.33
		Second derivative	(256, 84.4485)	96.67	100.00
		CARS	(147.0334, 84.4485)	93.33	90.00
		SPA	(48.5029, 147.0334)	96.67	96.67
	Set 2/OPW 3	PCA loadings	(147.0334, 256)	96.67	100.00
		Second derivative	(147.0334, 84.4485)	96.67	96.67
		CARS	(256, 147.0334)	92.22	96.67
		SPA	(27.8576, 147.0334)	96.67	96.67
	Set 3/OPW 1	PCA loadings	(9.1896, 147.0334)	97.78	90.00
		Second derivative	(147.0334, 27.8576)	97.78	93.33
		CARS	(147.0334, 256)	97.78	100.00
		SPA	(147.0334, 27.8576)	100.00	100.00
	Set 3/OPW 2	PCA loadings	(84.4885, 48.5029)	98.89	100.00
		Second derivative	(147.0334, 16)	98.89	100.00
		CARS	(147.0334, 91.1111)	91.11	86.67
		SPA	(256, 48.5029)	98.89	96.67

^a^ OPW 2 means the optimal wavelengths were selected by sample set 2; ^b^ OPW 3 means the optimal wavelengths were selected by sample set 3; ^c^ OPW 1 means the optimal wavelengths were selected by sample set 1.

**Table 5:** The results of the SVM models built by using the full spectra of sample set 3 as the calibration set and the untransferred and transferred full spectra of sample sets 1 and 2 as the prediction sets.

Pretreatment	Sample	Correctly Classified Samples/Total Healthy Samples	Correctly Classified Samples/Total Infected Samples	Total Prediction Accuracy (%)
Untransferred	Set 1	8/45	35/45	47.78
	Set 2	51/60	58/60	90.83
Transferred	Set 1	42/45	43/45	94.44
	Set 2	59/60	55/60	95.00

**Table 6:** The results of the different calibration models using combined sample sets.

Sample Sets	Models	Parameters	Calibration Accuracy (%)	Prediction Accuracy (%)
Combined Sets 1, 2, and 3	PLS-DA	10	92.74	92.68
	SVM	(256, 3.0314)	97.58	95.12
	KNN	3	87.10	90.24
	SIMCA	(20, 20)	80.24	84.15
